# Association between Long Noncoding RNA ANRIL Expression Variants and Susceptibility to Coronary Artery Disease

**DOI:** 10.22088/IJMCM.BUMS.7.1.1

**Published:** 2018-02-10

**Authors:** Mohsen Yari, Sara Bitarafan, Mohammad Ali Broumand, Zahra Fazeli, Mahnoosh Rahimi, Sayyed Mohammad Hossein Ghaderian, Reza Mirfakhraie, Mir Davood Omrani

**Affiliations:** 1 *Department of Medical Genetics, School of Medicine, Shahid Beheshti University of Medical Sciences, Tehran, Iran.*; 2 *Department of * *Molecular Pathology, Tehran Heart Center, Tehran University of Medical Sciences, Tehran, Iran.*; 3 *Department of Bioinformatics and Genomics* *, Pharmacogenetic Research Center, Simple LIMS, San Diego, CA, USA.*; 4 *Urogenital Stem Cell Research Center, Shahid Beheshti University of Medical Sciences, Tehran, Iran.*

**Keywords:** Coronary artery disease, atherosclerosis, long noncoding RNA, ANRIL, chromosome 9p21

## Abstract

Animal cells possess thousands of long non-coding (lnc) RNAs, such as antisense noncoding RNA in the INK4 locus (*ANRIL*), which have regulatory roles in the cells’ molecular mechanisms, including X-chromosome inactivation, and developmental processes. These lnc RNAs are known to influence the extensive spectrum of age-related disorders. Accordingly, there is evidence for the role of these lnc RNAs in cardiovascular diseases, particularly coronary artery diseases (CAD). The aim of this study was to assess whether the expression of the lnc RNA *ANRIL* was associated with a susceptibility to CAD by evaluating the expression level of the two transcripts of *ANRIL*. Peripheral blood was taken from fifty patients affected by CAD and relative expression of *ANRIL* was determined by Real-Time PCR assay. The obtained data indicated that the *EU741058* transcript expression level significantly decreased in CAD patients in comparison with the healthy individuals (P= 0.001). Furthermore, there was no significant association between the *NR_003529* transcript expression, and CAD risk in Iranian patients (P=0.751). Our results suggest that the expression level of the *EU741058* transcript of *ANRIL* may be implicated in CAD development, creating a predictive biomarker for CAD patients in future.

Cardiovascular disease (CVD) is known to be one of the main causes of human death worldwide (1). In 2012, the World Health Organization (WHO) has reported that ischemic heart disease has been mentioned as the first leading cause of mortality in the world (2). A cohort study on the Iranian population indicated that there was a high incidence of cardiovascular disease (CVD), and mortality in both sexes in Iran (3). Recently, genome-wide association studies (GWAS) have demonstrated that single nucleotide polymorphisms (SNPs) on chromosome 9p21 (Chr9p21) affect susceptibility to CVD, and coronary artery disease (CAD) in Caucasians and other populations (4-7). The chromosome region 9p21 has been reported to be an important susceptibility locus for several multifactorial diseases including CAD, ischemic stroke, aortic aneurysm and type 2 diabetes mellitus (8).

Long non-coding RNAs (lncRNAs) have been implicated in many important biological mechani-sms, in particular imprinting, histone-code regula-tion, gene regulation, and cell proliferation. Several studies have indicated that the lncRNA expression level is associated with manifesting some disorders including atherosclerosis (9-11). Antisense noncoding RNA in the INK4 locus (*ANRIL*) is a lncRNA with no identified open-reading frame. It is located on 9p21 chromosome region and is transcribed in the antisense orientation by the RNA polymerase II. This gene contains 21 exons and spans a region of almost 126.3 kb (12-15). *ANRIL* was spliced into different linear isoforms, and most of them were polyadenylated. *ANRIL* splicing variants have been known to regulate their neighbor tumor suppressors CDKN2A/B through epigenetic mechanisms (13, 16, 17).

Functional analysis of the 9p21.3 region has revealed that there might be a functional enhancer that influences the expression of *ANRIL* variants, *NR_003529* and *EU741058* (15). The previous studies showed that there was a significant association between risk alleles of *ANRIL* gene and risk of atherosclerosis (12, 18). Furthermore, the expression of *ANRIL* variants have been shown to be different between patients affected by atherosclerosis and healthy individuals (17). The methylation of the *ANRIL* target, *p15*^INK4b^, has been found to be associated with the expression of the EU741058 variant of the *ANRIL* gene in CAD patients (19). In the present study, we aimed to assess whether *ANRIL* expression variants were associated with the susceptibility to CAD in Iranian patients. The results obtained from this study could improve our understanding of the molecular mechanisms involved in the manifestion of CAD.

## Materials and methods

**Study population**

A total of fifty patients affected by CAD and fifty healthy individuals were recruited from the Department of Cardiology, Tehran Heart Center. In summary, a huge CAD was characterized as the vicinity of 50% luminal width narrowing in the left anterior descending artery, left circumflex vein, right coronary supply route, and their primary branches. The left primary trunk stenosis was considered as a two-vessel sickness. The seriousness of coronary atherosclerosis was further classified as a 1-, 2-or ≥3-vessel ailment as per the number of coronary vessels with critical stenosis (20, 21). The status of disease was determined by angiography. The demographic and biochemical characteristics were recorded for all studied individuals. All patients in this study signed a written consent. The present study has been approved by the Ethics Committee of Shahid Beheshti University of Medical Sciences, and Tehran Heart Center.

**RNA isolation and cDNA synthesis**

The peripheral venous blood was taken in the morning from patients fasting after midnight, and the total RNA was extracted by QIAamp RNA Blood Mini kit (Qiagen, Germany). After determining the concentration of RNA, the cDNA synthesis was carried out using a QuantiTect Reverse Transcription kit (Qiagen, Germany).

**Quantitative reverse-transcript polymerase chain reaction (qRT-** **PCR** **)**

The quantification of the relative expression was performed in triplicate for each sample. The beta actin was used as the reference gene to normalize the expression level of the *ANRIL* gene. The sequence of primers and probe were presented in Table 1. The expression level was determined using Premix Ex TaqTM (Takara Biotechnology, Tokyo, Japan) according to the manufacture’s guidelines. An initial denaturation was performed at 95 °C for 30 s followed by 45 cycles of 95 C for 10 s, and 60 C for 30 s. The PCR products were confirmed using a 2% agarose gel.

**Statistical analysis**

All statistical analyses were performed using SPSS 14.0 (SPSS Inc, Chicago, IL, USA), and REST software (Germany, 2009). A value of P<0.05 was considered to be statistically significant. The relative expression was calculated with the pfaffl formula. The receiver-operating-characteristics (ROC) curve was depicted to determine the predictive value of the *NR_003529* and *EU741058* variants in the detection of CAD. The area under the ROC curve was used to evaluate its overall diagnostic accuracy.

## Results

The demographic and biochemical characteris-tics of patients did not show a significant difference compared with the studied healthy individuals (Tables 2 and 3). However, most of the individuals of the present study were urban residents (Table 3). The study population comprised 68 males and 32 females with the mean age of 53 years (Table 2). The most common associated clinical condition in suspected patients with CAD was hypertension which was present in 46% of the study population while the least common one was smoking which represented 16% of the patients The expression analysis of the *ANRIL* transcripts including EU (exon1-5) and NR (exon 17-18) indicated that the *EU741058* variant was downregulated in the CAD patients as compared with healthy controls (P=0.001) (Figure 2). No significant difference was observed for *NR_003529* expression in the Iranian patients affected by CAD (P= 0.751). The ROC curve analysis also indicated that the sensitivity of *EU741058* and *NR_003529* for predicting CAD was 82% and 64%, respectively. The specificity of these transcripts was estimated at 69% and 47%, respectively. As shown in Figure 3, the area under the ROC curve demonstrated that these two transcripts might be used with caution as a biomarker of CAD detection (*EU741058*; 0.81 and *NR_003529*; 0.71)

**Fig. 1:**
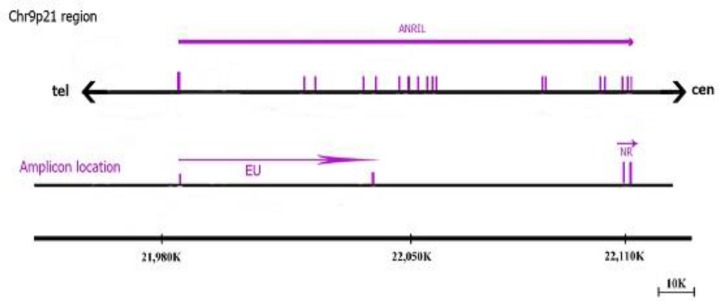
Map of ANRIL transcripts and positions of its 20 exons on chromosome 9p21. ANRIL: antisense non-coding RNA in the INK4 locus; cen: centromere; tel: telomere

**Table 1:** Primer and probe sequences used for quantitative RT-PCR

Gene (Ref.)	Primer and/or probe sequence (5’→3’)
*ANRIL exon 1-5* *EU741058 *(13)	Forward: TGCCGGAGCTGTCGACCC Reverse: CTTTGATCTCTGCTGTTGAATCAGAATG Probe: 6FAM-CGGCCTGGCGCCGGACTAGTGTC-TAMRA
*ANRIL exon 17-18* *NR_003529 *(13)	Forward: CAGAGCAATTCCAGTGCAAG Reverse: GATTTGCAAAAACAGCTG Probe: 6FAM-CTGCTACATGGAGGCTAGGGCCAGAGTCA-TAMRA
*β-Actin *(13) ENST00000331789	Forward: CCTGGCACCCAGCACAAT Reverse: GCCGATCCACACGGAGTACTT Probe: 6FAM-ATCAAGATCATTGCTCCTCCTGAGCGCA-TAMRA

**Table 2:** Clinical characteristics of Iranian patients with CAD and the healthy individuals under study

**Characteristics **	**CAD (n=50)**	**Control (n=50)**	**p-value**
Age	53.84	51.62	0.077
Male	35 (70%)	33 (66%)	0.415
Smoking	8 (16%)	9 (18%)	0.500
Hypertension	25 (50%)	21 (42%)	0.274
Diabetes mellitus	15 (30%)	10 (20%)	0.178
TG (mg/dL)	171.711	162.808	0.890
LDL-C (mg/dL)	112.400	101.255	0.887
HDL-C (mg/dL)	40.222	43.106	0.159
The use of Statin	35	28	0.323

**Table 3:** Socio-demographic characteristics of the studied population

Characteristics	Status	
	Affected by CAD	Healthy individuals (control)
Sex (male/female %)	66/34	70/30
Residence (urban/rural %)	60/40	92/8
Age (mean± SD years)	53.84±7.32	51.62±8.87

**Fig. 2:**
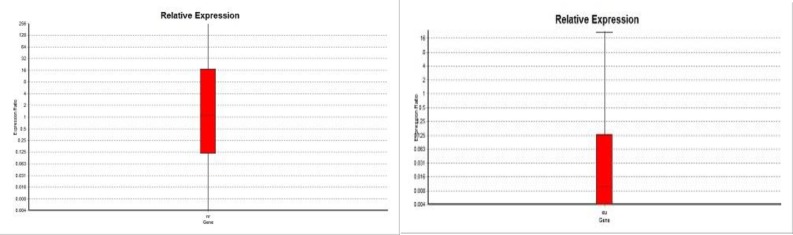
Histograms showing the relative expression of different transcripts in CAD patients as compared with healthy controls. A: NR transcript; B: EU transcript. As observed, there was no significant association between NR_003529 transcript and risk of CAD (A) and the expression of EU741058 transcript decreased in the patients with CAD (B).

## Discussion

In recent years, a new class of non-protein coding transcripts has been considered within the molecular mechanisms affecting different diseases, particularly age-related disorders. The identification of their role in the disease process could increase our understanding of the pathogenesis of these diseases. In the present study, the expression of *ANRIL* was reported in the the Iranian patients with CAD for the first time. The aim was to ascertain whether the expression of the *ANRIL* transcripts involved in the manifestion of coronary artery disease in the Iranian patients. As a non coding RNA, *ANRIL* serves different tasks in DNA damage response, epigenetic regulation of *p15*^INK4b^, controlling the cell cycle checkpoints, apoptosis and DNA repair (22, 23). The study performed by Zhuang et al. (2012) has revealed that the methylation of the *p15*^INK4b^ promoter was associated with the up-regulation of the *ANRIL* expression in the Chinese patients affected by CAD (19). Our finding in the previous study indicated that the methylation pattern of the *p15*^INK4b^ promoter in the CAD patients was slightly different from the healthy individuals (21). In the present study, the expression analysis indicated that the expression of the *EU741058 *transcript was decreased in the peripheral blood of the Iranian CAD patients as compared with the healthy study participants (P=0.001). Besides, there was no association between the expression of *NR_003529* and the susceptibility to CAD in the Iranian patients (Figure 2). Our results were inconsistent with the data obtained by Zhuang et al., suggesting a different etiology of CAD in the Iranian and Chinese populations. Some previous studies have revealed that the Iranians had a phylogenetic origin different from the Chinese population (24). Furthermore, the frequency of some demographic and clinical characteristics was different between these two studied populations. Several risk factors have been identified to play a role in the susceptibility to CAD. They included age, gender, smoking, hypertension, high blood cholesterol and diabetes mellitus (25).

**Fig. 3:**
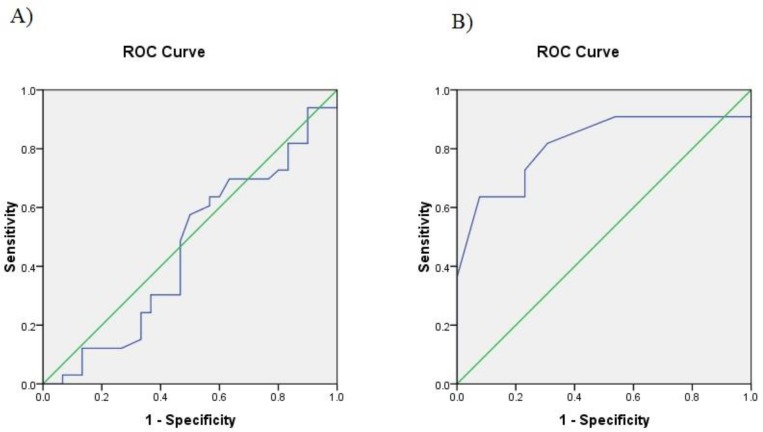
ROC curve of *ANRIL* expression by Real time PCR. ROC curve could be used to predict the susceptibility to CAD in terms of sensitivity and specificity. A: *NR_003529* variant, B: *EU741058* variant.

Some other studies presented a significant association between the risk of atherosclerosis and the expression of the ANRIL variants (12). Our finding showed that the expression of some variants of ANRIL (EU741058) could influence the susceptibility of CAD in the Iranian patients. However, there was some discrepancy between the expression level of ANRIL in the present study and the latter one, suggesting the role of different factors in the pathophysiology of CAD and atherosclerosis. Furthermore , Kotake et al. (2011) demonstrated that the Ras induction could inhibit the expression of ANRIL (26). Ras, an important signaling molecule involved in atherogenic stimuli, has several roles in the vascular smooth muscle cell senescence, and inflammation (27). Therefore, it is possible that the downregulation of ANRIL in the present study plays a role in the etiology of CAD through the vascular senescence.

The variants of Chr9p21.3 have revealed a risk for heart-related diseases (1). Although Dehghan et al. demonstrated that some single nucleotide polymorphisms (SNPs) located at chromosome 9p21 were not associated with a susceptibility to coronary heart diseases (CHDs), some studies indicated that there was a significant correlation between the SNPs of Chr9p21 with atherosclerosis (12, 28). Recently Ghochin et al. found two SNPs in CDKN2B-AS (*ANRIL *locus) in a subgroup of north Iranian population with significantly higher prevalence (29). This discrepancy observed in the association of SNPs located on Chr9p21 with heart disease indicated that it is likely that the environmental factors play an important role in the susceptibility to CHD. Therefore, different lifestyle could explain the inconsistent results obtained from *ANRIL* expression in our population as compared with other studies.

In summary, the results obtained from the present study suggested that the *ANRIL* expression was dysregulated in the patients with CAD. It seems that the etiology of CAD was complex, and several factors could influence the pathophysiology of this disorder in the different populations. Furthermore, the data of the ROC analysis indicated that the expression analysis of *ANRIL* might be a suitable prognosis biomarker of CAD. The study of additional genes harboring long non coding (lnc) RNAs and their downstream targets could provide a better understanding of the disease mechanisms, and the right predictable biomarkers.

## Conflict of interest

The authors report no conflicts of interest.
